# Real-world data of fulvestrant as first-line treatment of postmenopausal women with estrogen receptor-positive metastatic breast cancer

**DOI:** 10.1038/s41598-021-83622-1

**Published:** 2021-02-19

**Authors:** I. Blancas, C. Olier, V. Conde, J. L. Bayo, C. Herrero, I. Zarcos-Pedrinaci, F. Carabantes, J. M. Baena-Cañada, J. Cruz, M. Ruiz-Borrego

**Affiliations:** 1 Oncology Department, Hospital Universitario Clínico San Cecilio and Medicine Department, Granada University, Avenida del Conocimiento s/n, 18006 Granada, Spain; 2 Oncology Department, Hospital Universitario Fundación Alcorcón, Calle Budapest, 1, 28922 Alcorcón, Madrid, Spain; 3 Oncology Department, Hospital Universitario Virgen de Las Nieves, Av. de las Fuerzas Armadas, s/n, 18014 Granada, Spain; 4 Oncology Department, Hospital Juan Ramón Jiménez, Ronda Exterior Norte s/n, 21005 Huelva, Spain; 5 Oncology Department, Hospital Provincial de Castellón, Castelló de La Plana, Av. del Dr. Clarà, 19, 12002 Castellón, Spain; 6 Oncology Unit, Hospital Costa del Sol, Km 187, 29603 Marbella, Málaga, Spain; 7 Oncology Department, Hospital Regional Universitario de Málaga, Av. de Carlos Haya, s/n, 29010 Málaga, Spain; 8 Oncology Department, Hospital Universitario Puerta del Mar and Instituto de Investigación E Innovación Biomédica de Cádiz (INiBICA), 11009 Cádiz, Spain; 9 Oncology Department, Hospital Universitario de Canarias, Carretera de Ofra, s/n, 38320 Santa Cruz de Tenerife, Spain; 10 Oncology Department, Hospital Universitario Virgen del Rocío, Av. Manuel Siurot, s/n, 41013 Sevilla, Spain

**Keywords:** Cancer, Breast cancer

## Abstract

Goals of endocrine therapy for advanced breast cancer (ABC) include prolonging survival rates, maintaining the quality of life, and delaying the initiation of chemotherapy. We evaluated the effectiveness of fulvestrant as first-line in patients with estrogen receptor (ER)-positive ABC with relapse during or after adjuvant anti-estrogenic therapy in real-world settings. Retrospective, observational study involving 171 postmenopausal women with ER-positive ABC who received fulvestrant as first-line between January 2011 and May 2018 in Spanish hospitals. With a median follow-up of 31.4 months, the progression-free survival (PFS) with fulvestrant was 14.6 months. No differences were seen in the visceral metastatic (14.3 months) versus non-visceral (14.6 months) metastatic subgroup for PFS. Overall response rate and clinical benefit rate were 35.2% and 82.8%. Overall survival was 43.1 months. The duration of the clinical benefit was 19.2 months. Patients with ECOG performance status 0 at the start of treatment showed a significant greater clinical benefit rate and overall survival than with ECOG 1–2. Results in real-world settings are in concordance with randomized clinical trials. Fulvestrant continues to demonstrate clinical benefits in real-world settings and appears be well tolerated as first-line for the treatment of postmenopausal women with ER-positive ABC.

## Introduction

Breast cancer (BC) is the most frequent type of carcinoma and leading cause of cancer-related mortality in women^1^. Incidence and survival rates vary considerably among countries around the world due to diverse factors such as population structure (age, race, ethnicity), lifestyle, environment, socioeconomic status, genetics, disease stage at diagnosis, and healthcare (mammography use, access to high-quality care)^2^. At diagnosis, most of patients present with early BC (EBC), however about 6–10% of cases have distant metastases^3^. Furthermore, approximately 30% of patients with EBC will develop metastatic BC (MBC) in the following months or years^4^. The expression of estrogen receptor (ER) and/or the progesterone receptor (PR) are used as prognostic factors in patients, and for predicting the response to adjuvant endocrine therapy, the main therapeutic option for ER-positive advanced breast cancer (ABC) patients^5,6^. In this line, ER-positive BC comprise approximately 70% of all BC cases^7^. Goals of endocrine therapy for ABC include prolonging survival rates, maintaining the quality of life, and delaying, as possible, the initiation of chemotherapy^8^. Despite the proven efficacy of the endocrine therapy in these patients, one in three cases will develop resistance and, thus, disease progression^9^. Recently, cyclin-dependent kinase 4/6 (CDK4/6) inhibitors combined with endocrine therapy have been recommended in first- or second-line treatment for patients with ER-positive/HER2-negative MBC^10^. However, there are still many patients who, due to comorbidities, elderly fragility or in order to avoid an added toxicity, could be suitable for receiving only hormonotherapy on this scenario. Thus, it seems relevant to know exactly what to expect from fulvestrant in first-line treatment of MBC with real-world data. Fulvestrant is a synthetic, selective ER antagonist that triggers the degradation of the ER protein^11^. Several phase 3 trials have demonstrated the efficacy and safety of fulvestrant, in monotherapy and in combination with other agents, for the treatment of ER-positive ABC. One of them is the international FALCON trial that compared the efficacy of fulvestrant with an aromatase inhibitor, anastrozole, in 462 ER-positive ABC patients who had not received previously an endocrine therapy^12^. Fulvestrant showed superior efficacy in terms of progression-free survival (PFS, 16.6 months; 95% confidence interval, 95% CI 13.8–21.0 months) than anastrozole (13.8 months, 95% CI 12.0–16.6 months), and similar rates of adverse events (AEs). Real-world evidence is an important step for completing data obtained from randomized clinical trials^13^. Therefore, the objective of the present study was to evaluate the effectiveness of fulvestrant as first-line for the treatment of patients with ER-positive MBC with relapse during or after the completion of adjuvant anti-estrogenic therapy in real-world settings.

## Material and methods

This retrospective, observational study involved postmenopausal women with ER-positive ABC who received fulvestrant as first-line between January 2011 and May 2018. A total of 11 centers across Spain participated in the study. Inclusion criteria were: Informed consent written and signed prior to any specific study of procedures (in the case of patients who died at the time of inclusion, there were no signed informed consent, so the researcher assumed responsibility for data protection and confidentiality); postmenopausal women, according to NCCN definition, i.e. those who underwent a previous bilateral oophorectomy, aged ≥ 60 years, or aged < 60 years and amenorrhea for ≥ 12 months in the absence of chemotherapy, tamoxifen, toremifene or ovarian suppression, as well as follicle-stimulating hormone and estradiol^14^; diagnosis of MBC with histological/cytological confirmation; positive hormone receptor status (ER-positive and/or PgR-positive) from primary or metastatic tumor tissue receptors; Eastern Cooperative Oncology performance status (ECOG PS) score of 0, 1 or 2 at the time of starting fulvestrant^15^; having experienced recurrence during or after completing the adjuvant anti-estrogenic therapy; and receiving fulvestrant 500 mg as first-line, i.e. subsequently to the recurrence. Exclusion criteria included: Patients who were not available for the signature date of informed consent (in the case of patients alive at the time of study inclusion) or later than the date of baseline study; having received fulvestrant previously; having received any other therapy (different to fulvestrant) after the recurrence; previous neoplasia (other than BC or treatment for basal or squamous cell carcinoma of the skin or cervical carcinoma in situ) unless it has been treated curatively with no evidence of disease in the last 5 years; and patients with HER2-positive BC (3-positive HER2 overexpression by immunohistochemistry or positive gene amplification by fluorescence in situ hybridization or similar techniques). The status of ER and PgR was evaluated by using an immunohistochemical analysis with antibodies against ER and PgR, respectively^16^. The classification of Luminal A and B was also established according to immunohistochemistry, i.e. luminal A (ER-positive, PgR-positive, HER2-neagtive, Ki67% ≤ 20), and luminal B (other cases)^16,17^. Patients were followed-up for a minimum period of 12 months were in accordance with Declaration of Helsinki and approved by the Ethics Committee of Hospital Universitario San Cecilio (Spain).

### Studied variables

The effectiveness of fulvestrant was determined in terms of PFS. The PFS was defined as the elapsed time between start of treatment with fulvestrant and disease progression or death, by any cause. Secondary endpoints included: overall survival (OS), overall response rate (ORR), duration of the response, clinical benefit rate (CBR), duration of the clinical benefit (DoCB), response to subsequent treatments, progression-free rate (PFR), safety, and tolerability. The OS was defined as the elapsed time between start of treatment with fulvestrant and death, by any cause. At the time of data collection, data from patients who were alive without progression were censored for the survival analysis on the date of their last follow-up. The ORR was calculated as the sum of complete response (CR) and partial response (PR); whereas CBR as CR, PR and stable disease (SD) persisting for ≥ 24 weeks. The PFR was defined as the proportion of patients who remained with no disease progression or death. The AEs were reported by using the Common Terminology Criteria for Adverse Events (CTCAE) V4.0. Primary endocrine resistance was defined as the relapse occurrence while on the first 2 years of adjuvant endocrine treatment; secondary endocrine resistance as the relapse occurrence while on adjuvant endocrine treatment but after the first 2 years; and hormone-sensitive patients as the relapse occurrence after 1 year of finishing 5 years on adjuvant endocrine treatment.

### Statistical analysis

Quantitative variables were expressed as median, interquartile range (IQR) or 95% CI; whereas qualitative ones as absolute and relative frequencies. Survival analyses were carried out by following Kaplan–Meier methodology. Comparison of survival curves between subgroups was performed using Log-rank tests. Comparison of variables between subgroups was carried out using parametric (T test or analysis of variance, ANOVA), or nonparametric (Mann–Whitney U or Kruskal–Wallis) tests, when appropriate. Statistical significance was established when *p* ≤ 0.05. All statistical procedures were performed using SAS software 9.4.

### Ethics approval

Procedures were in accordance with Declaration of Helsinki and approved by the Ethics Committee of Hospital Universitario San Cecilio (Spain).

### Consent to participate

All patients signed the informed consent to participate before being included in the study.

## Results

### Study population

**Figure 1:**
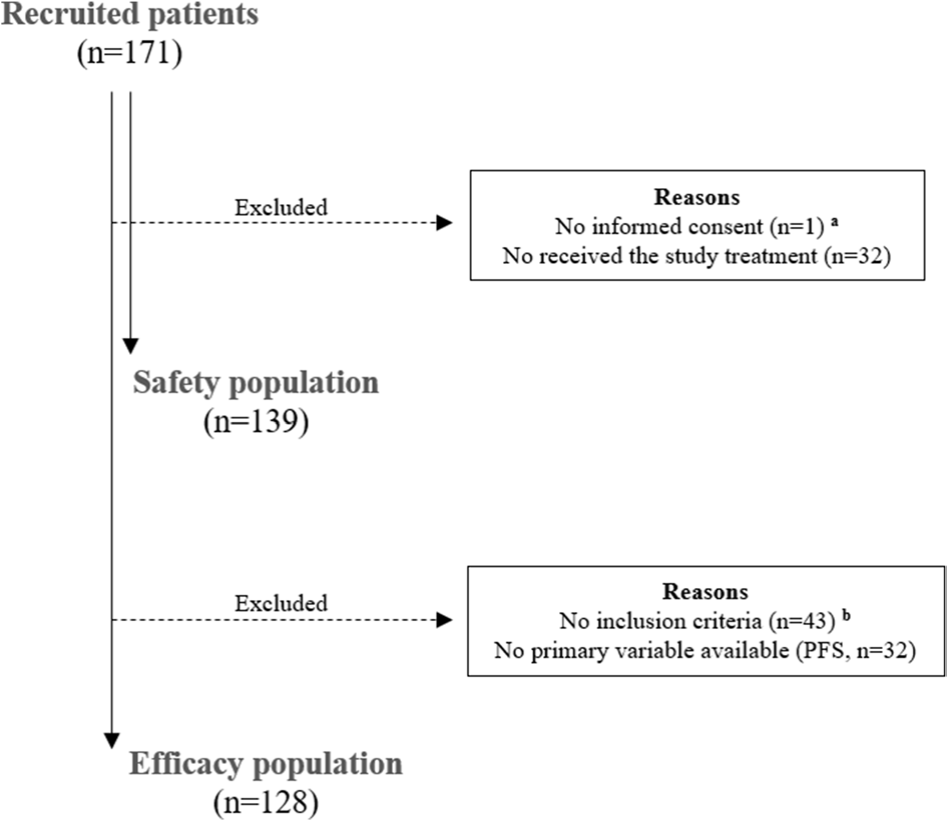
Patient flowchart. (**a**) Informed consent written and signed prior to any specific study of procedures. In the case of patients who died at the time of inclusion, there were no signed informed consent, so the researcher assumed responsibility for data protection and confidentiality when recording the data; (**b**) Patients who experienced relapse during or after the end of adjuvant anti-estrogenic therapy and received first-line treatment with Fulvestrant 500 mg per month during the study period (started treatment with Fulvestrant January 1, 2011 to May 30, 2018.

A total of 171 patients were recruited in the study; however, 128 fulfilled the inclusion criteria. A patient flowchart is shown in Fig. 1. Patients had a median age of 69.2 years (IQR, 59.0–78.9 years) at the time of study inclusion. Ductal carcinoma (76.6% of patients) was the most predominant histological type of the EBC; predominantly of grade 2 (moderately differentiated, 59.8% of patients). Demographic and clinical characteristics of patients are shown in Table 1. At the time of EBC diagnosis, 68.8% of patients were ER-positive, 78.9% PgR-positive, 68.6% showed a low Ki67 proliferation index (≤ 20%). None of the patients presented with de novo metastatic disease. The median time from diagnosis of early disease to ABC was 6.8 years (IQR, 3.7–10.5 years). Biopsy for metastatic location was performed in 47.7% of patients. A total of 51.6% of patients developed metastases in non-visceral locations (41.5% in bones, 10.2% in local relapse). At the time of starting treatment with fulvestrant, 93.0% of patients had ECOG PS 0–1. The median duration of the treatment with fulvestrant was 14.0 months (IQR, 6.9–26.6 months).

**Table 1:** Demographic and clinical characteristics of patients.

	Patients (N = 128)*
Age at study inclusion, median years (IQR)	69.2 (59.0–78.9)
**Early breast cancer**	
Time from diagnosis to study inclusion, median years (IQR)	11.1 (7.6–15.6)
Histological type, n (%)	
Ductal carcinoma	98 (76.6)
Lobular carcinoma	20 (15.6)
Mucinous carcinoma	3 (2.3)
Others	7 (5.5)
Cancer grade, n (%)	
N available	117
Grade 1 (Differentiated)	25 (21.4)
Grade 2 (Moderately differentiated)	70 (59.8)
Grade 3 (Poorly differentiated)	22 (18.8)
Hormone receptors, n (%)	
ER-positive	88 (68.8)
PgR-positive	101 (78.9)
Ki67 proliferation index, n (%)	
N available	121
Low (≤ 20%)	83 (68.6)
High (> 20%)	38 (31.4)
Unknown	7
Type of cancer, n (%)	
N available	121
Luminal A	61 (50.4)
Luminal B	60 (49.6)
Unknown	7
Adjuvant treatments, n (%)	
Chemotherapy	128 (100.0)
Hormonal	128 (100.0)
Radiotherapy	128 (100.0)
Advanced breast cancer**	
Time from diagnosis to study inclusion, median years (IQR)	4.0 (2.5–6.0)
Time from early to advanced disease, median years (IQR)	6.8 (3.7–10.5)
Hormone receptors – on biopsy, n (%)	
N available	61
ER-positive	38 (62.3)
PgR-positive	43 (70.4)
Unknown	67
Ki67 proliferation index, n (%)	
N available	56
Low (≤ 20%)	33 (58.9)
High (> 20%)	23 (41.1)
Unknown	72
Type of cancer, n (%)	
N available	85
Luminal A	41 (48.2)
Luminal B	44 (51.8)
Unknown	43
Metastasis location, n (%)	
Visceral	62 (48.4)
Bone	53 (41.4)
Local relapse	13 (10.2)
Previous treatments in the adjuvant setting, n (%)	
N available	127
Aromatase inhibitor	74 (58.3)
Aromatase inhibitor + tamoxifene	27 (21.3)
Tamoxifene	26 (20.5)
Unknown	1
**Treatment with fulvestrant**	
Time from diagnosis of ABC and start of treatment, median months (IQR)	0.9 (0.3–1.8)
ECOG PS at start of treatment, n (%)	
0	66 (51.6)
1	53 (41.4)
2	9 (7.0)
Duration of the treatment, median months (IQR)	14.0 (6.9–26.6)
**Endocrine resistance**	
Primary endocrine resistance, n (%)	24 (18.8), 114 (89.1), and 24 (18.8) ***
Secondary endocrine resistance, n (%)	90 (70.3), 114 (89.1), and 104 (81.2) ***
Hormone-sensitive patients, n (%)	14 (10.9), 14 (10.9), and 104 (81.2) ***

*IQR* interquartile range, *EBC* early breast cancer, *ER* estrogen receptor, *PgR* progesterone receptor, *ECOG PS* Eastern Cooperative Oncology Group performance status. *Available n for each variable is 128, unless otherwise indicated. **None of the patients presented with de novo metastatic disease. Metastatic information is from the same patients with early breast cancer. ***First n (%) for primary endocrine resistance, secondary endocrine resistance and hormone-sensitive patients calculated separately; second n (%) for the combination of primary with secondary endocrine resistance, and hormone-sensitive patients; and third n (%) for primary endocrine resistance, and the combination of secondary endocrine resistance with hormone-sensitive patients.

### Effectiveness outcomes

**Figure 2:**
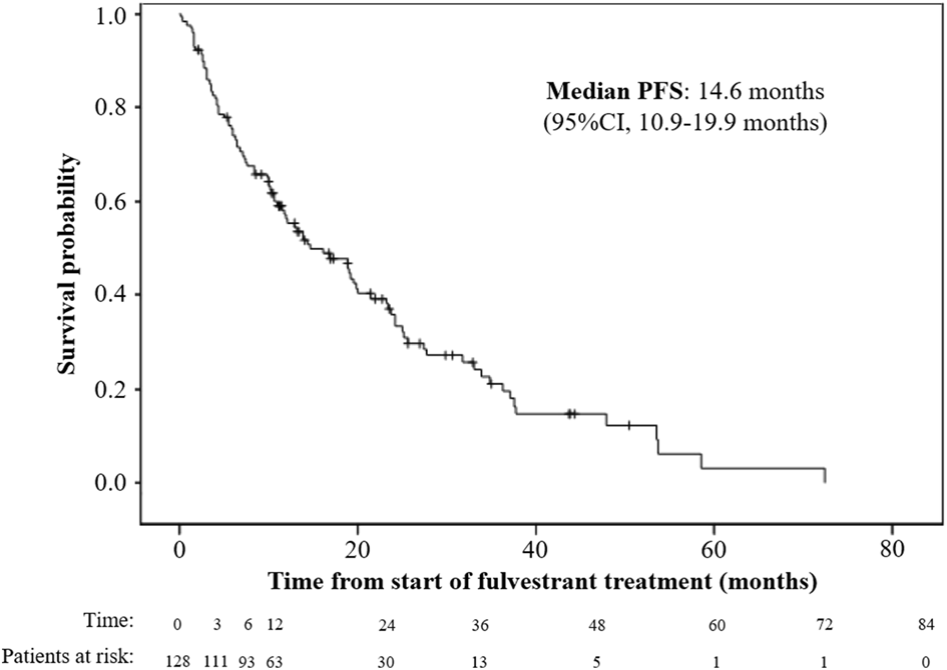
Progression-free survival from the start of fulvestrant treatment.

**Figure 3:**
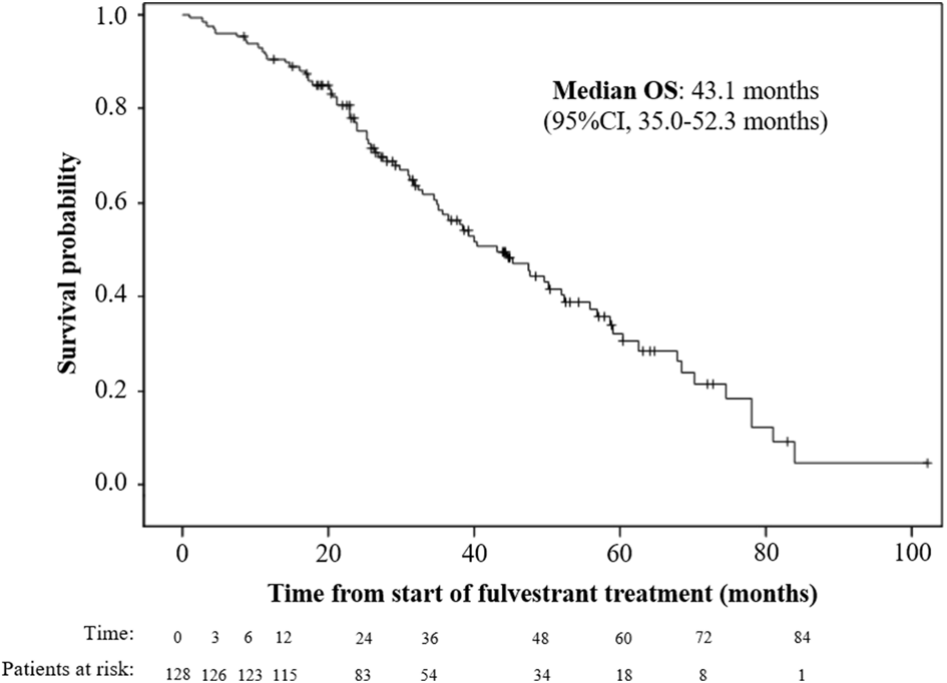
Overall survival from the start of fulvestrant treatment.

With a median follow-up period of 31.4 months, the median PFS with fulvestrant was 14.6 months (95% CI 10.9–19.9 months; Fig. 2). Effectiveness outcomes considering subgroups of patients are shown in Table 2. No differences in PFS were found regarding type of cancer, Ki67 proliferation index, ECOG PS at the start of treatment with fulvestrant, metastasis location, adjuvant treatments, and endocrine resistance. However, patients ER-positive/ PgR-positive in ABC (on biopsy, median 23.2 months; 95% CI 11.5–25.2 months) showed a significant longer PFS than ER-positive /PgR-negative (median 16.1 months; 95% CI 1.6–21.5 months; *p* = 0.048). The CBR was 82.8% (95% CI 75.1–88.9%). Significant differences in CBR were found regarding: hormone receptors (at EBC; 86.1% for ER-positive/ PgR-positive versus 70.4% for ER-positive /PgR-negative; *p* = 0.054); hormone receptors (at ABC, on biopsy; 95.4% for ER-positive / PgR-positive versus 53.8% for ER-positive /PgR-negative; *p* = 0.001); and ECOG PS at the start of treatment (90.9% for ECOG 0 versus 74.2% for ECOG 1–2; *p* = 0.012). The median of DoCB with fulvestrant was 19.2 months (95% CI 14.0–24.1 months). The ORR was 35.2% (95% CI 26.9–44.1%). No significant differences in DoCB or ORR were found regarding subgroups (data not shown). The median OS from the start of the fulvestrant treatment was 43.1 months (95% CI 35.0–52.3 months; Fig. 3). Patients with ECOG PS 0 at the start of treatment (median 47.6 months; 95% CI 35.7–70.2 months) showed a significant longer OS than those with ECOG PS 1–2 (median 36.5 months; 95% CI 26.9–50.1 months; *p* = 0.024). No differences in OS were found regarding type of cancer, hormone receptor, Ki67 proliferation index, metastasis location, adjuvant treatments, and endocrine resistance. The PFR was 26.6% (95% CI 19.1–35.1%). No differences in PFR were found regarding subgroups (data not shown). A total of 96 patients initiated a subsequent treatment after disease progression with fulvestrant: chemotherapy (46 patients, 47.9%); hormonal therapy (46 patients, 47.9%), and radiotherapy (4 patients; 4.2%). After this treatment, 78.1% were free of progression: 73.9% in patients with chemotherapy, 75.0% with radiotherapy, and 82.6% with hormonal therapy. A total of 42 patients reported a second subsequent treatment: Chemotherapy (64.3%), hormonal therapy (26.2%), and radiotherapy (9.5%).

**Table 2:** Efficacy outcomes considering subgroups of patients.

		Progression-free survival			Clinical benefit rate		Overall survival		
	N	Median	95% CI	*p* value	n (%)	*p* value	Median	95% CI	*p* value
**Type of cancer (EBC)**									
Luminal A	61	16.9	11.5–23.6	0.570	54 (88.5)	0.198	43.1	35.0–60.3	0.340
Luminal B	60	13.2	7.4–21.5		48 (80.0)		40.0	26.9–58.6	
**Type of cancer (ABC, on biopsy of the metastasis)**									
Luminal A	25	18.8	8.3–27.4	0.208	23 (92.0)	0.249	49.6	25.3–83.8	0.733
Luminal B	26	16.9	3.4–21.5		20 (76.9)		52.3	23.0–N.R	
**Hormone receptors (EBC)**									
ER-positive / PgR-positive	101	18.1	11.7–23.2	0.328	87 (86.1)	0.054	44.4	35.0–55.8	0.260
ER-positive /PgR-negative	27	10.9	6.8–27.7		19 (70.4)		34.5	20.3–78.0	
**Hormone receptors (ABC, on biopsy of the metastasis)**									
ER-positive / PgR-positive	43	23.2	11.5–25.2	0.048	41 (95.4)	0.001	49.6	35.7–68.4	0.224
ER-positive /PgR-negative	13	16.1	1.6–21.5		7 (53.8)		29.9	16.9–58.6	
**Ki67 proliferation index (EBC)**									
Low (≤ 20%)	83	14.6	10.6–19.9	0.748	72 (86.8)	0.274	44.4	25.6–58.6	0.371
High (> 20%)	38	14.3	5.5–24.3		30 (80.0)		43.1	35.0–55.8	
**Ki67 proliferation index (ABC, on biopsy of the metastasis)**									
Low (≤ 20%)	33	16.1	8.3–24.1	0.888	20 (87.0)	1.000	40.0	23.9–40.7	0.692
High (> 20%)	23	19.8	5.8–23.6		28 (84.9)		52.3	25.4–N.R	
**ECOG PS at the start of treatment**									
0	66	14.6	10.9–25.0	0.760	60 (90.9)	0.012	47.6	35.7–70.2	0.024
1–2	62	14.0	7.7–19.9		46 (74.2)		36.5	26.9–50.1	
**Metastasis location**									
Visceral	62	14.3	10.7–24.1	0.901	49 (79.0)	0.272	38.0	29.9–47.6	0.343
Non-visceral	66	14.6	10.2–23.2		57 (86.4)		49.6	35.0–59.1	
**Previous treatments in the adjuvant setting**									
Aromatase inhibitor	74	16.1	10.2–19.9	0.758	59 (79.7)	0.557	36.5	30.9–52.3	0.692
Aromatase inhibitor + tamoxifene	27	23.2	12.0–27.4		24 (88.9)		55.8	29.9–70.2	
Tamoxifene	26	12.1	6.0–31.8		22 (84.6)		47.4	25.4–58.6	
**Resistance 1**									
Primary endocrine resistance	24	12.0	10.0–25.0	0.551	19 (79.2)		31.7	23.9–47.6	
Secondary endocrine resistance	90	14.6	10.0–23.2		75 (83.3)	0.931	45.3	35.0–58.6	0.223
Hormone-sensitive patients	14	19.5	6.8–47.9		12 (85.7)		52.0	25.3–74.5	
**Resistance 2**									
Primary/secondary endocrine resistance	114	14.3	10.6–19.8	0.275	94 (82.5)	1.000	40.3	32.9–50.1	0.509
Hormone-sensitive patients	14	19.5	6.8–47.9		12 (85.7)		52.0	25.3–74.5	
**Resistance 3**									
Primary endocrine resistance	24	12.0	10.0–25.0	0.795	19 (79.2)	0.561	31.7	23.9–47.6	0.092
Secondary endocrine resistance/hormone-sensitive patients	104	16.9	10.7–23.2		87 (83.7)		47.4	36.5–58.6	

*EBC* early breast cancer, *ABC* advanced breast cancer, *ER* estrogen receptor, *PgR* progesterone receptor, *ECOG PS* Eastern Cooperative Oncology Group performance status, *N.R.* not reached.

### Safety profile

The number of evaluable patients for safety analyses was 139. Of them, 93 patients (66.9%) experienced a total of 553 AEs.12 patients (8.6%) reported 14 serious AEs no related with the treatment: respiratory/thoracic/mediastinal disorders (4 cases), gastrointestinal disorders (2 cases), hepatobiliary disorders (2 cases), infections and infestations (2 cases), musculoskeletal and connective tissue disorders (2 cases), injury/poisoning/procedural complications (1 case), and metabolism and nutrition disorders (1 case). The most prevalent AEs were: musculoskeletal and connective tissue disorders (53 patients, 38.1%), asthenia (48, 34.5%), injection site reactions (30, 21.6%), nausea (18, 12.9%), decreased appetite (18, 12.9%), and diarrhea (15, 10.8%). 32 AEs (5.8% of the total) were causal relationship: Injection site reaction or hypersensitivity (7); asthenia (7); gastrointestinal disorders (6); musculoskeletal and connective tissue disorders (3); blood and lymphatic system disorders (2); respiratory- thoracic and mediastinal disorders (2); headache (1); fatigue (1); tachycardia (1); nail disorder (1); and decreased appetite (1) (Table 3). One patient died because of a serious AE (grade 5 hemoptysis) not related with the fulvestrant.

**Table 3:** Incidence of most frequent adverse events.

Adverse event	Number of patients (%)
Musculoskeletal and connective tissue disorders	53 (38.1)
Asthenia	48 (34.5)
Injection site reactions	30 (21.6)
Nausea	18 (12.9)
Decreased appetite	18 (12.9)
Diarrhea	15 (10.8)
Headache	13 (9.4)
Dyspnea	13 (9.4)
Peripheral neuropathy	11 (7.9)
Palmar-plantar erythrodysesthesia syndrome	11 (7.9)
Vomiting	10 (7.2)
Abdominal pain—upper	8 (5.8)
Neutropenia	7 (5.0)
Constipation	7 (5.0)
Rash	7 (5.0)
Hot flush	7 (5.0)

## Discussion

The efficacy and safety of fulvestrant is mainly based on results from 3 pivotal randomized clinical trials^12,18–22^. In the aforementioned phase III FALCON study, involving women who did not receive a previous endocrine therapy, the CBR was 78% with fulvestrant and 74% with anastrozole^12^. The median DoCB was also numerically longer with fulvestrant (22.1 months; 95% CI 18.5–24.9 months versus 19.1 months; 95% CI 16.5–20.5 months)^12^. The OS was not calculated due to insufficient follow-up at the time of data cut-off, however a lower percentage of women who died was observed in the fulvestrant group (29%) than with anastrozole (32%). The most frequent AE associated to fulvestrant were arthralgia (38 patients, out of 228 evaluable ones for safety, 17%), hot flush (26 patients, 11%), fatigue (26 patients, 11%), nausea (24 patients, 11%), and back pain (21 patients, 9%). The Fulvestrant First-Line Study Comparing Endocrine Treatments (FIRST) phase II trial compared the efficacy and safety of fulvestrant 500 mg and anastrozole 1 mg as first-line endocrine therapy in 205 postmenopausal women with ABC^18–20^. With fulvestrant, ORR (36%) and CBR (72.5%) were similar than with anastrozole (35.5% and 67.0%, respectively). Although the median DoCB had not been reached at primary data cut-off, the tendency favored fulvestrant, with respect to anastrozole^18^. A subsequent follow-up analysis (when 79.5% of patients had discontinued the study treatment) revealed a significant longer time to progression with fulvestrant (23.4 months) than anastrozole (13.1 months), i.e. 34% reduced the progression risk^19^. Moreover, the OS was improved with fulvestrant (54.1 months versus 48.4 months with anastrozole)^20^. Regarding safety, gastrointestinal disturbances (28 patients, out of 101 evaluated ones, 27.7%), joint disorders (14 patients, 13.9%), and hot flashes (13 patients, 12.9%) were the most prevalent AEs related to fulvestrant^18^. Finally, the phase III Comparison of Faslodex Recurrent or Metastatic Breast Cancer (CONFIRM) trial compared the efficacy and safety of fulvestrant 250 and 500 mg in 736 postmenopausal women with ABC^21,22^. Fulvestrant 500 mg was used in both first- and second- lines in CONFIRM population. The ORR and CBR with fulvestrant 250 mg were 10.2 and 39.6 months, respectively; and 9.1 months and 45.6 months with fulvestrant 500 mg^21^. Fulvestrant 500 mg showed a significant longer PFS (6.5 months) versus 250 mg (5.5 months). The median OS was 22.3 and 26.4 months for fulvestrant 250 and 500 mg, respectively^22^. Most prevalent grade ≥ 3 AEs with fulvestrant 250 mg were: joint disorders (8 patients, out of 374 evaluable ones, 2.1%), thromboembolic events (4 patients, 1.1%), and ischemic cardiovascular disorders (3 patients, 0.8%); whereas with fulvestrant 500 mg were joint disorders (8 patients, out of 361 evaluable, 2.2%), and gastrointestinal disturbances (8 patients, 2.2%)^21^.

Results from our present real-world study are in concordance with pivotal trials^12,18–22^: CBR (82.8%, slightly higher than 78% in FALCON trial)^12^, DoCB (19.2 months, slightly reduced than 22.1 months in FALCON trial); PFS (14.6 months, similar to 16.6 months in FALCON trial), and OS (43.1 months, slightly reduced than 54.1 months in FIRST trial^21^. The combination of a CDK4/6inhibitor (such as palbociclib, ribociclib or abemaciclib) with endocrine therapy is the standard treatment for ER-positive/HER2-negative MBC^10^. Recent studies have demonstrated that the combination of a CDK4/6 inhibitor and fulvestrant improves OS in patients with hormone receptor-positive, HER 2-negative MBC, either after failure of endocrine therapy (and irrespective of menopausal status), and as first- or second-line therapy in postmenopausal women^23,24^. Our present study provides real-world efficacy data from fulvestrant (a high CBR and PFS of 14 months) that can be useful for patients who are not candidate for receiving a CDK4/6 inhibitor, due to possible added toxicity of the inhibitor, other comorbidities, elderly fragility, or that the patient is not able of undergoing the frequent clinical and hematologic controls required for CDK4/6 inhibitors. Indeed, ESMO has recommended for the management of MBC patients during the COVID-19 pandemic that, when combining CDK4/6 inhibitors with endocrine therapies, it is needed to consider risks of neutropenia, and that the patient requires close monitoring of symptoms of infection^25,26^. And so, considering the option of postponing a line of CDK4/6 inhibitors, for bone only, low burden, de novo metastatic disease, particularly in the elderly. Therefore, nowadays, the results from our study are relevant because an important group of patients could benefit from fulvestrant in first-line of MBC treatment.

On the other hand, there are no currently clinical tools that predict which patients may benefit from diverse endocrine therapies^27^. The CONFIRM and FIRST trials also evaluated PFS according to the characteristics of patients; however, the treatment efficacy was consistent across subgroups^19–21^. By contrast, FALCON study revealed that visceral involvement showed a significant differentiation in PFS (fulvestrant versus anastrozole)^12^. In patients receiving fulvestrant, PFS was 22.3 months (95% CI 16.6–32.8 months) for those with non-visceral disease, and 13.8 months (95% CI 11.0–16.5 months) with visceral involvement. In our real-world study, visceral disease was not associated with a differential effectiveness of fulvestrant. Nevertheless, ECOG PS 0 at the start of treatment was associated with greater CBR and OS, compared with ECOG 1–2.

Regarding the safety profile, reported AEs were in line with observed previously in clinical trials^12,18–22^. Most frequent AEs included those concerning the musculoskeletal and connective tissue, administration site, and gastrointestinal disorders. The AEs did not raise any major concerns. The main limitation of the study derived from its retrospective design; providing only available data.

## Conclusion

Results in real-world settings are in concordance with previously observed in randomized clinical trials. Moreover, fulvestrant continues to demonstrate clinical benefits in real-world settings, and appears well tolerated as first-line for the treatment of postmenopausal women with ER-positive ABC.

## Data Availability

The datasets generated during and/or analyzed during the current study are available from the corresponding author on reasonable request.
